# Carbenoxolone inhibits TRPV4 channel‐initiated oxidative urothelial injury and ameliorates cyclophosphamide‐induced bladder dysfunction

**DOI:** 10.1111/jcmm.13100

**Published:** 2017-02-28

**Authors:** Xiling Zhang, Shan Gao, Masayoshi Tanaka, Zhen Zhang, Yanru Huang, Takahiko Mitsui, Manabu Kamiyama, Schuichi Koizumi, Jianglin Fan, Masayuki Takeda, Jian Yao

**Affiliations:** ^1^ Division of Molecular Signaling Department of Advanced Biomedical Research University of Yamanashi Yamanashi Japan; ^2^ China Medical University Shenyang China; ^3^ Department of Neuropharmacology University of Yamanashi Yamanashi Japan; ^4^ Department of Urology University of Yamanashi Yamanashi Japan; ^5^ Department of Molecular Pathology Interdisciplinary Graduate School of Medicine and Engineering University of Yamanashi Yamanashi Japan

**Keywords:** carbenoxolone, bladder dysfunction, TRPV4, oxidative stress, p38 activation, calcium

## Abstract

Carbenoxolone (CBX) is a clinically prescribed drug for the treatment of digestive ulcer and inflammation. It is also a widely used pharmacological inhibitor of several channels in basic research. Given that the overactivity of several channels, including those inhibitable by CBX, underlies bladder dysfunction, we tested the potential therapeutic application and mechanism of CBX in the treatment of voiding dysfunction. In a mouse model of cystitis induced by cyclophosphamide (CYP), CBX administration prevented the CYP‐elicited increase in bladder weight, oedema, haemorrhage, and urothelial injury. CBX also greatly improved micturition pattern, as manifested by the apparently decreased micturition frequency and increased micturition volume. Western blot results showed that CBX suppressed CYP‐induced increase in protein carbonyls, COX‐2, and iNOS. Further analysis using cultured urothelial cells revealed that acrolein, the major metabolite of CYP, caused protein oxidation, p38 activation, and urothelial injury. These effects of acrolein were reproduced by TRPV4 agonists and significantly prevented by antioxidant NAC, p38 inhibitor SB203580, TRPV4 antagonist RN‐1734, and CBX. Further studies showed that CBX potently suppressed TRPV4 agonist‐initiated calcium influx and subsequent cell injury. CBX attenuated CYP‐induced cystitis *in vivo* and reduced acrolein‐induced cell injury *in vitro*, through mechanisms involving inhibition of TRPV4 channels and attenuation of the channel‐mediated oxidative stress. CBX might be a promising agent for the treatment of bladder dysfunction.

## Introduction

Voiding dysfunction, as manifested by urinary frequency, urgency, and incontinence, occurs in many types of bladder disorders, including interstitial cystitis, overactive bladder and obstruction of the urinary tract and urination difficulties arisen from neurological conditions or spinal cord injury. Although these symptoms are not life‐threatening, they severely affect the patients’ quality of life 1, 2, 3, 4. Currently, the available therapeutic options for voiding dysfunction are still limited and unsatisfactory. It is highly desirable to understand the molecular mechanisms involved in the initiation and development of these symptoms and to find more effective therapeutic approaches.

The molecular mechanisms for frequent urination in humans remain elusive. Studies from animal experiments have shown a critical involvement of several membrane channels, including TRPV1, TRPV4, connexin (Cx), pannexin, ATP‐gated P2X3 and P2X7 receptor channels, as well as L‐type voltage‐gated Ca^2+^ channels 5, 6, 7, 8, 9, 10, 11, 12, 13, 14. TRPV4 is a Ca^2+^‐permeable cation channel, highly expressed in mouse, rat and human bladder urothelium 10, 15, 16. It is activated by a wide range of stimuli, including cell swelling, heat, mechanical stimulation, endocannabinoids, arachidonic acid and 4alpha‐phorbol esters. Once activated, it mediates stimuli‐evoked Ca^2+^ influx and ATP release. In the bladder, the mechanosensitive TRPV4 channel plays a role in sensing the normal filling state of the bladder. Genetic disruption or pharmacological inhibition of TRPV4 increases functional bladder capacity and improves CYP‐induced bladder dysfunction 10, 16, 17, 18. The membrane Cx and pannexin channels also play an important role in the regulation of bladder function. These two channel‐forming proteins share many similarities in structure and function. Cxs form gap junctions that mediate the transfer of ions, metabolites and second messengers between contacting cells, whereas membrane channels formed by pannexin allow the exchange of small molecules between cell and extracellular environment. Both Cx and pannexin channels have been shown to be pivotally involved in the regulation of cell function and tissue homeostasis. They participate in the transmission and propagation of intercellular calcium (Ca^2+^) signalling that is required for the coordinated multicellular function, including the bladder filling and emptying activity 18, 19, 20. Indeed, several studies have shown that Cx43 channels take part in the control of voiding activity 7, 8. Suppression of Cx43 with AMPK agonists or genetic disruption of Cx43 greatly improved CYP‐induced cystitis and voiding dysfunction 5. Pannexin channels can also be activated by a variety of stimuli and release ATP. It has been implicated in the induction of Cx43 expression, inflammatory responses and bladder overactivity in a mouse model of multisclerosis and a rat model of voiding dysfunction initiated by activation of urothelial P2Y6 receptors 6, 21. These studies indicate that TRPV4, Cx and pannexin channels are an integral part of the mechanism involved in the control of bladder function. The overactivity of these channels is closely related to symptoms of overactive bladder. In this context, inhibition of these channels with the pharmacological inhibitors may help ameliorate bladder symptoms 5, 6, 9, 22. Indeed, a newly characterized TRPV4 inhibitor HC‐067047 has been shown to be promising in the treatment of mouse voiding dysfunction 9. However, the drug has not been clinically tested. There has been a great interest in finding clinically prescribed drugs that have the potential to suppress the activities of these channels.

CBX is a glycyrrhetinic acid derivative used for the treatment of peptic, oesophageal and oral ulceration and inflammation in the clinic 21. It is also an extensively used pharmacologic inhibitor of Cx and pannexin channels in the basic research 22, 23. Besides, CBX also suppresses oxidative and inflammatory responses in several different pathological situations 24, 25, 26. These properties of CBX made us speculate that it might be exploited to treat voiding dysfunction. This study was designed to test this hypothesis.

Herein, we present our findings that CBX attenuated CYP‐induced cystitis *in vivo* and reduced acrolein‐elicited cell injury *in vitro*, possibly through mechanisms involving inhibition of TRPV4 channels and the channel‐mediated oxidative urothelial injury. CBX might be a promising drug for treatment of bladder dysfunction.

## Materials and methods

### Reagents

COX‐2 antibody was purchased from Cayman Chemical (Ann Arbor, MI, USA). Anti‐iNOS was bought from Enzo Life Sciences (NY, USA). Anti‐GAPDH, horseradish peroxidase‐conjugated anti‐rabbit IgG and phospho‐p38 mitogen‐activated protein kinase (MAPK) (Thr180/Tyr182) antibodies were obtained from Cell Signaling Technology (Danvers, MA, USA). CYP, carbenoxolone disodium (CBX), 4α‐Phorbol 12,13‐didecanoate (4α‐PDD), GSK1016790A, RN‐1734, foetal bovine serum (FBS), trypsin/EDTA, antibiotics, and all other chemicals were from Sigma‐Aldrich (Tokyo, Japan).

### Animals

Adult female C57BL/6J mice aged 10–12 weeks, weighing 20–25 g, were used for all experiments. Mice were housed in containment facilities of the animal centre and fed with food and water in an air‐conditioned room with a 12 hrs light/dark cycle. All the animal experimental procedures were reviewed and approved by the animal care and use committee of Yamanashi University.

### CYP‐induced mouse cystitis

A total of 56 adult female C57BL/6J mice were divided into four groups: control, CYP cystitis, CBX control and CBX‐treated group. Each group consisted of 3 ~ 4 mice. The same experiments were repeated 3 times. For induction of CYP cystitis, mice were intraperitoneally injected with 150 mg/kg CYP in a volume of 200 μl saline. The control mice received the same volume of saline. For CBX control and treatment, mice were intraperitoneally administrated with 50 mg/kg CBX at 12‐hrs interval for three times before injection of saline or CYP, respectively 5, 27, 28. At the time as described in figure legends, mice were subjected to the metabolic cage for micturition patter evaluation or bladders were taken for histochemical or Western blot analysis.

### Micturition analysis

Mouse micturition pattern was monitored using a metabolic cage system as described previously 5. Briefly, mice were kept in metabolic cages in a soundproof room at 25°C temperature under 12‐hrs light and 12‐hrs dark cycle. Each mouse was provided with free access to food and water. After being adapted to the environment for 2 days in the cages, the behaviours of voiding and water intake before and after different drug administration were monitored with the special mesh device and recording equipment, which were placed under the cages and linked to a computer. The collected data were analyzed using the PowerLab LabChart software (AD Instruments, Colorado Springs, CO). The cage system allows us to monitor micturition pattern of four mice simultaneously. One representative mouse from each group was recorded during one batch of experiments. The experiments were repeated 4 times.

### Western blot analysis

Proteins extraction and western blot analysis were performed as described previously 5. Briefly, extracted proteins were loaded onto 10% SDS–polyacrylamide gels and electrotransferred onto polyvinylidene difluoride membranes. After blocking with 5% non‐fat dry milk or 3% BSA in PBS, the membranes were incubated with primary antibody overnight at 4°C. After washing, the membranes were probed with horseradish peroxidase‐conjugated anti‐rabbit or anti‐mouse IgG, and the bands were visualized using Chemi‐Lumi One L (Nacalai Tesque, Kyoto, Japan). The chemiluminescent signal was captured with a Fujifilm luminescent image LAS‐1000 analyzer (Tokyo, Japan). GAPDH was used as an internal loading control.

### Assessment of protein oxidation

The protein carbonylation was analyzed using OxyBlot Protein Oxidation Detection Kit (EMD Millipore, Billerica, MA, USA) as reported previously 5. Briefly, protein lysate was prepared by suspending mouse bladder tissue in lysis buffer (8 mol/l urea, 1 mmol/l dithiothreitol, 1 mmol/l ethylenediaminetetraacetic acid, 50 mmol/l Tris‐HCl, pH 8.0) or cultured cells in sample buffer (62.5 mM Tris‐HCl, 2% SDS, 10% glycerol), supplemented with proteinase inhibitor cocktail (Nacalai Tesque) and 50 mM DTT. Five‐microlitre protein samples at the amount of 15–20 μg were transferred into Eppendorf tubes and mixed with 5 μl of 12% SDS and 10 μl of 1 × DNPH (2,4‐dinitrophenylhydrazine) solution to denature and derivatize the proteins, respectively. After reaction for 15 min. at room temperature, 7.5 μl neutralization solution was added and the samples were subjected to Western blot analysis.

### Histochemistry assay

Mice bladder tissues were fixed with 4% paraformaldehyde at room temperature for 72 hrs, followed by 30% sucrose for an additional 24 hrs, and then embedded in molten paraffin. Three‐micrometre‐thick section was cut with a microtome. The sections were haematoxylin and eosin (H&E) stained for tissue histology.

### Primary culture of urothelial cells

Primarily, cultured urothelial cells were obtained from the bladders of C57BL/6J mice, using the method as previously reported 10. Briefly, the whole bladders were taken from anesthetized mice. The bladders were inverted by pushing the dome downward through the bladder neck with a blunt 18‐gauge needle and incubated with 0.05% trypsin‐EDTA for 30 min. at 37°C. After that, urothelial cells were collected, centrifuged and resuspended in keratinocyte serum‐free medium (KSFM, Invitrogen, Life Technologies, Carlsbad, CA, USA) containing 5 ng/ml epidermal growth factor, 50 μg/ml bovine pituitary extract, 30 ng/ml cholera toxin (all from Invitrogen) and 0.5% penicillin/streptomycin/antibiotic antimycotic solution (ABAM; Sigma‐Aldrich, Carlsbad, CA,USA). The cells were seeded in 96‐ or 24‐well plates precoated with collagen (0.01%; Sigma‐Aldrich) and cultured in a humidified atmosphere of 5% CO_2_/95% air at 37°C. Cells after 72 hrs of cultivation were used for experiments.

### Modulation of TRPV4 activity

TRPV4 channels were activated by exposing cells to TRPV4 agonist 4α‐PDD or GSK1016790A at the concentration of 10 μM or 100 nM, respectively. In contrast, it was inhibited by treatment of cells with 10 μM TRPV4 antagonist RN‐1734.

### Lactate dehydrogenase (LDH) assay

Cell cytotoxicity was quantitatively assessed using a commercial kit (LDH Cytotoxicity Detection Kit, TaKaRa) following the protocol provided by the manufacturer 29. Briefly, cells were seeded into a 96‐well plate at the density of 15,000 per well in 100 μl DMEM/F12 supplemented with 0.5% FBS. After stimulation, a 25‐μl supernatant was collected and incubated with the same volume of assay buffer at room temperature for 30 min. The intensity of blue colour formation was measured using a spectrometer at 490 nm. LDH release was calculated and expressed as the percentage of the total release. The culture media was used as the background control, while cell lysate obtained from 2% Triton X‐100 was taken as 100% release.

### Propidium iodide (PI) staining

Cell viability was determined with the Cellstain Double Staining Kit (Dojindo, Tokyo, Japan). Briefly, cells were seeded into a 96‐well plate and stimulated with the indicated chemicals for the described time. Afterwards, cells were exposed to 4 μM PI for 10 min. The viable and dead cells are distinguished by their fluorescent colour under the immunofluorescence microscope. PI enters cells through damaged cell membranes of dead cells and interacts with nucleic acids, emitting red fluorescence. Images of red cells were captured under the identical capture settings using Olympus CCD camera attached to the immunofluorescent microscope (IX71S1F‐2; Olympus, Tokyo, Japan).

### Measurement of intracellular Ca^2+^

For measurement of Ca^2+^, renal tubular epithelial cell line, NRK‐52E, was used. Cells were washed once with a balanced salt solution (BSS; 8.76 g/l NaCl, 5 mM KCl, 1.8 mM CaCl_2_, 1.2 mM MgCl_2_, 5.96 g/l HEPES and 1.8 g/l glucose, pH 7.4) and loaded with 5 μM fluorescent Ca^2+^ indicator fura‐2‐acetoxymethyl ester (Molecular Probes) for 45 min**.** Afterwards, cells were either treated with CBX for 30 min. or left untouched before exposing to TRPV4 agonist 4α‐PDD or GSK1016790A. Measurement of intracellular Ca^2+^ was performed by ratiometric imaging with fura‐2 at 340 nm and 380 nm. F340/F380 was calculated and acquired with an imaging processing system (IP‐Lab, Scanalytics Inc., Rockville, MD, USA). Changes in the ratio (Δ) were calculated by subtracting basal values from peak values.

### Statistical analysis

Values are expressed as mean ± S.E. Comparison of two groups was made by Student's *t*‐test. For multiple comparisons, one‐way analysis of variance followed by Dennett's test was employed. Both analyses were performed using Microsoft Excel (Microsoft, Redmond, WA, USA). *P* < 0.05 was considered statistically significant.

### Study approval

All animal experiments were approved by the animal care and use committee of Yamanashi University.

## Results

### CBX attenuates CYP‐induced cystitis

**Figure 1:**
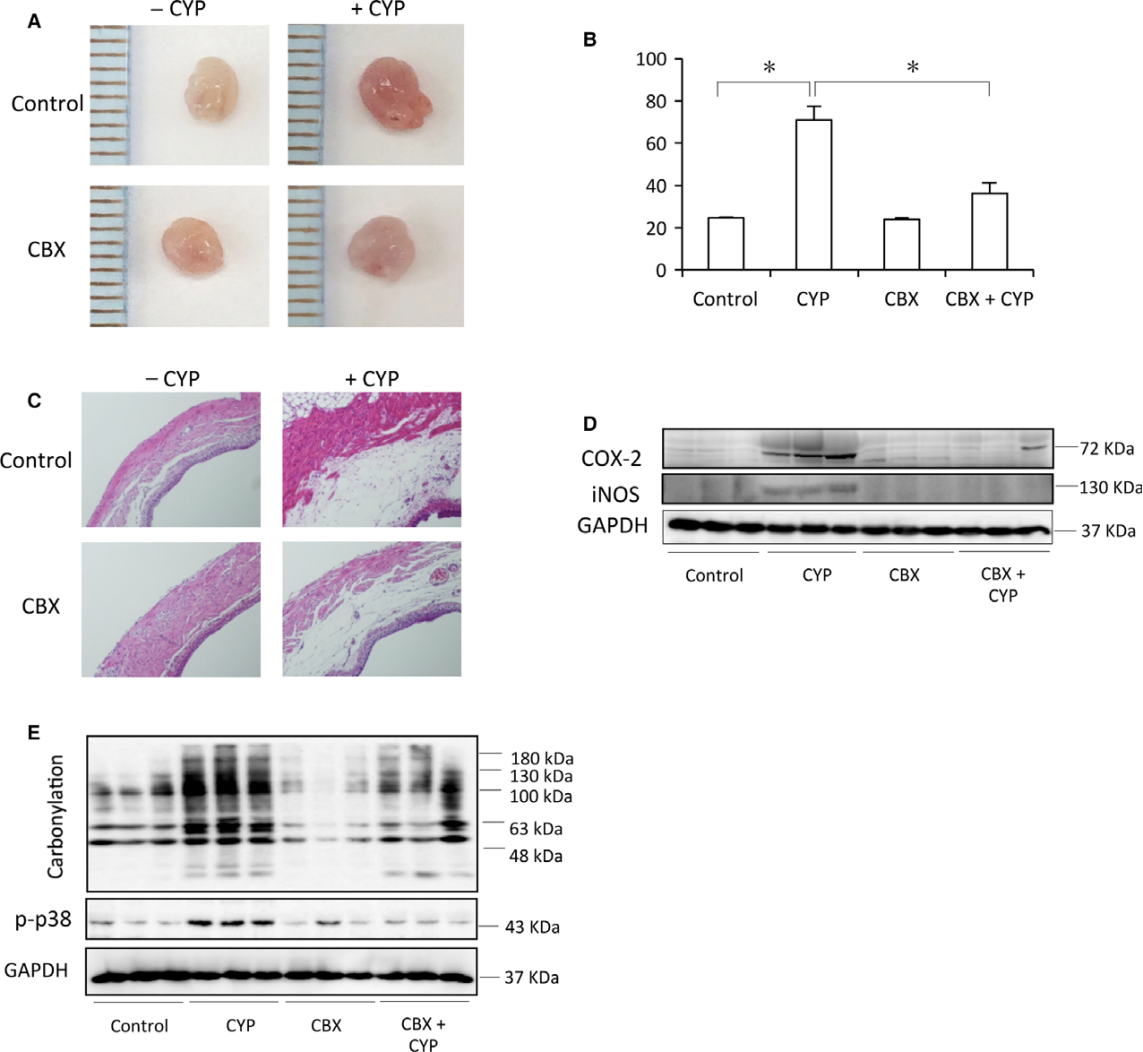
CBX attenuates CYP‐induced cystitis. Mice were divided into four groups: control, CYP cystitis, CBX control and CYP cystitis treated with CBX. Each group consisted of 3~4 mice and the same experiments were repeated three times. For CBX treatment, mice were pre‐treated with CBX (intraperitoneally injection, 50 mg/kg) at 12‐hrs interval for three times before administration of CYP (150 mg/kg) for an additional 24 hrs. (**A**) Representative images of mouse bladder from control and CBX‐treated groups. Note the obvious congestion, enlargement and haemorrhage in the CYP‐treated bladder. (**B**) Bladder weight in different groups. Data shown are mean ± S.E. from total of ten mice from three separate experiments. *P < 0.01. (**C**) Representative histological bladder sections from differently treated mice. (**D**,** E**) The bladder was taken 24 hrs (**D**) or 6 hrs (**E**) after CYP injection, respectively. Bladder proteins were extracted and subjected to Western blot analysis for COX‐2, iNOS, protein carbonylation, phospho‐P38 and GAPDH. Data presented are representative of three sets of independent experiments, in which each group had 3 to 4 mice.

To evaluate the potential therapeutic effects of CBX on CYP‐induced cystitis, we randomly divided mice into four groups: control, CYP cystitis, CBX control and CBX‐treated CYP cystitis. Each group consisted of 3 ~ 4 mice. The same experiments were repeated 3 times. Figure 1A shows the changes in bladder structure and function in mice treated with or without CBX. CYP administration caused bladder injury, as evidenced by the appearance of bladder haemorrhages, congestion and oedema. The bladder weight was significantly increased, being more than two times heavier than that of control (Fig. 1A and B). Histochemical staining of bladder sections revealed that CYP administration resulted in urothelial injury and lamina propria oedema, as manifested by the detachment of urothelial layer and the markedly increased thickness of lamina propria layer (Fig. 1C). A set of high magnification images of the histochemical changes has been shown in the Figure S1. Western blot analysis showed that CYP administration increased the levels of protein carbonyls, COX‐2 and iNOS, indicative of an existence of bladder oxidation and inflammation (Fig. 1D and E). In the mouse treated with CBX, however, all these pathological changes were greatly attenuated (Fig. 1A–E).

**Figure 2:**
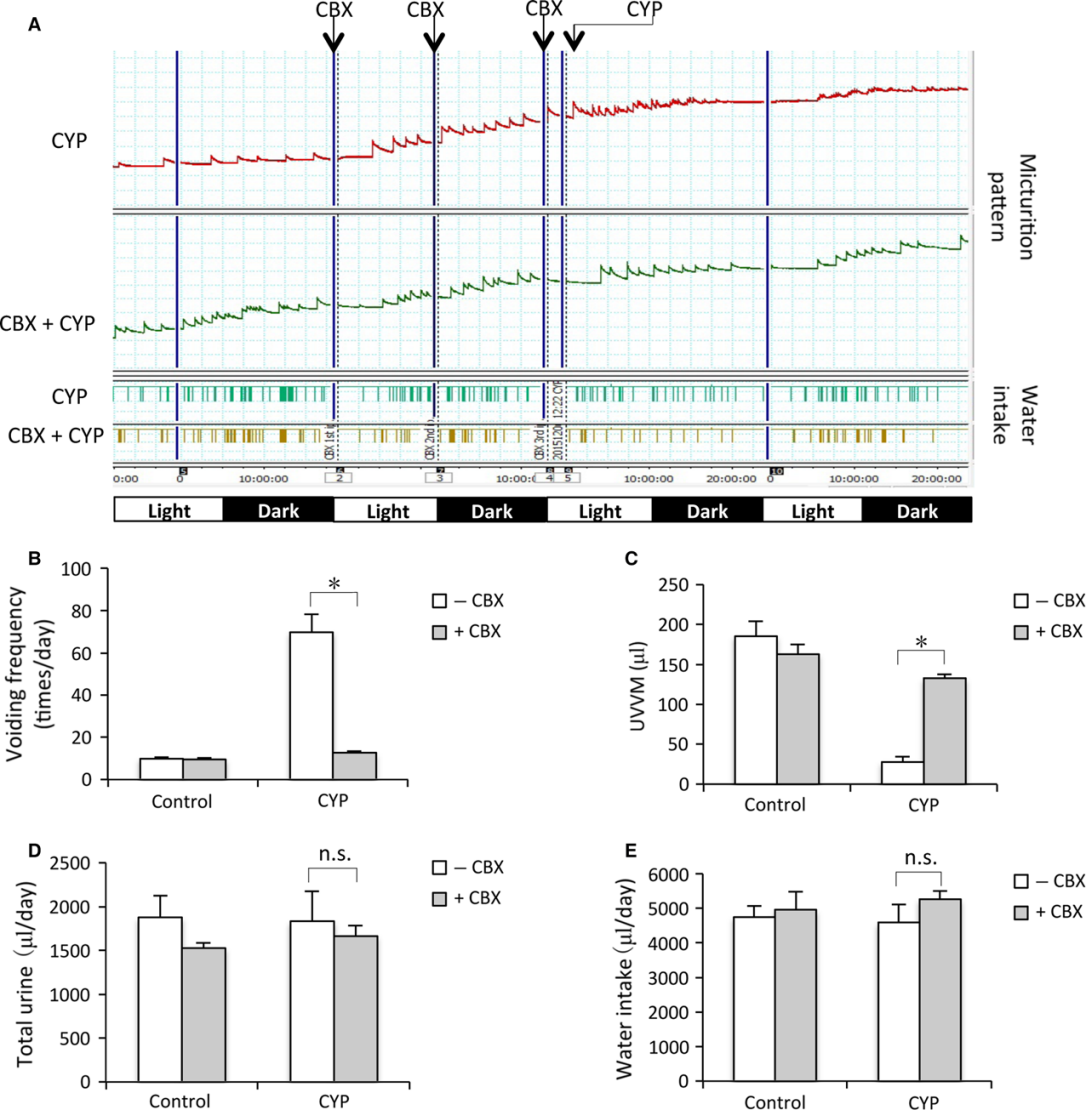
CBX ameliorates voiding dysfunction in CYP‐induced mouse cystitis. (**A**) Micturition patterns in different groups. Mice were treated the same as above. Micturition patterns were recorded. On the *X*‐axis, the black squares indicate dark periods (9:00 p.m. to 9:00 a.m.) and the white squares indicate light periods (9:00 a.m. to 9:00 p.m.). Data shown are representative of four separate experiments (*n* = 4). (**B**–**E**) UVVM, voiding frequency, total urine volume/day and water intake in different groups. A total of 4 mice were used for each group. Data are expressed as mean ± S.E., *n* = 4. *P < 0.01, n.s. = no significance.

Functional analysis of micturition pattern using metabolic cage shows that CYP administration caused pollakiuria, as indicated by the obviously increased voiding frequency and reduced urine volume voided per micturition (UVVM). CBX treatment, however, improved all the symptoms; the mice retained relatively normal voiding pattern (Fig. 2A–C), and the total urine volume per day and water intake between the treated and untreated mice were not greatly different (Fig. 2D and E). These results indicate that CBX attenuates CYP‐induced changes in bladder structure and function.

### CBX reduces acrolein‐induced cell injury through suppression of oxidative stress and p38 activation

**Figure 3:**
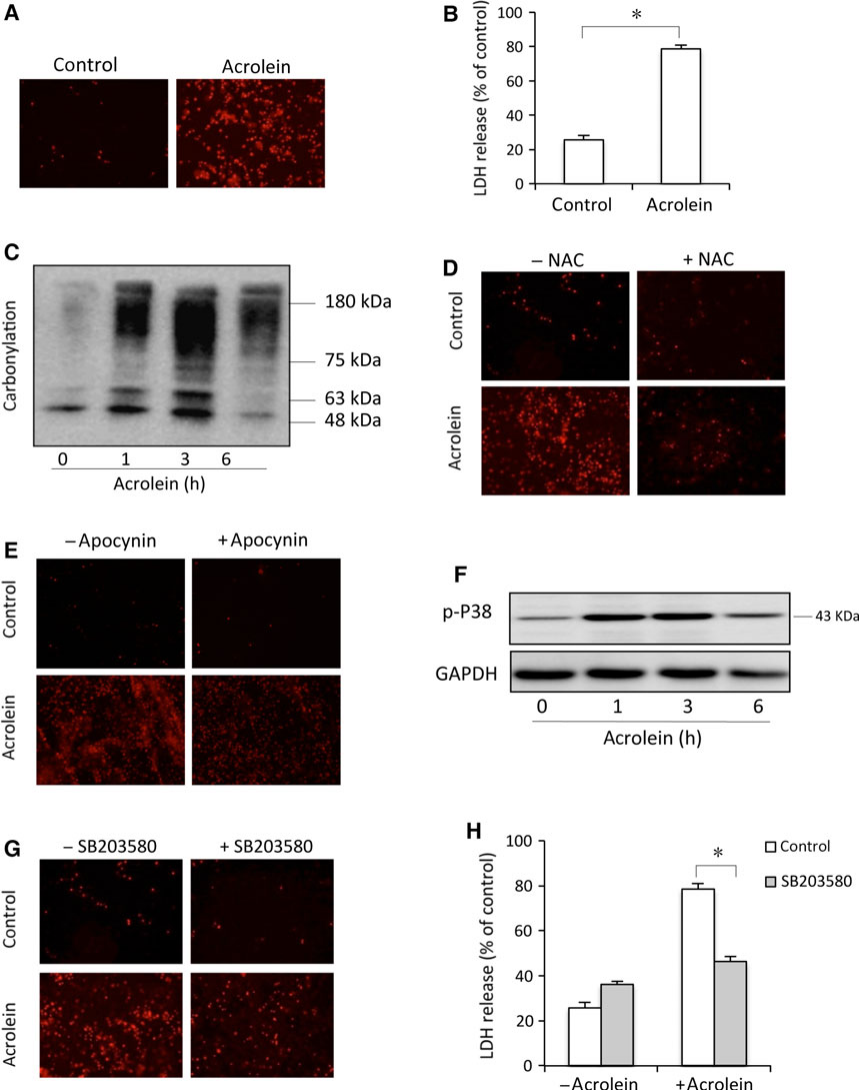
Acrolein elicits oxidative urothelial injury. (**A**–**B**) Primarily cultured urothelial cells were exposed to 100 μM acrolein for 12 hrs. Afterwards, the cells were subjected to PI staining (**A**) and cell supernatants were collected for LDH assay (**B**). Data are expressed as mean ± S.E. (*n* = 3). *P < 0.01. (**C**) Induction of protein carbonylation by acrolein. Primarily cultured urothelial cells were exposed to 100 μM acrolein for the indicated time intervals. Then, cell lysates were subjected to OxyBlot™ Protein Oxidation Detection Kit for carbonylated proteins. (**D**,** E**) Effects of antioxidant and NADPH oxidase inhibitor on cell viability. Urothelial cells were exposed to 100 μM acrolein for 12 hrs in the presence or absence of 10 mM NAC (**D**), or 10 mM apocynin (**E**). After that, cells were subjected to PI staining. (**F**) Induction of P38 phosphorylation by acrolein. Urothelial cells were incubated with 100 μM acrolein for the indicated time. Cellular lysates were subjected to Western blot analysis for phosphorylated P38. (**G**,** H**) Effects of P38 inhibitor SB230850 on cell viability. Urothelial cells were incubated with 100 μM acrolein for 12 hrs in the presence or absence of 20 μM P38 inhibitor SB203580. Afterwards, cells were stained with PI (**G**) and cell supernatants were collected for LDH assay (**H**). Data are expressed as mean ± S.E. (*n* = 3). *P < 0.01.

Several previous studies indicate that CYP‐induced cystitis is mediated by its metabolic product acrolein 21, 30, 31. To explore the mechanisms behind CYP‐induced urothelial cell injury, we cultured urothelial cells and analyzed acrolein‐induced cell injury *in vitro*. Figure 3A and B show that acrolein caused urothelial cell injury, as evidenced by the increased number of PI‐positive red cells, and elevated level of extracellular LDH. Before cell death, acrolein induced an elevation in protein carbonyls, indicative of an induction of oxidative stress (Fig. 3C). Treatment of cells with antioxidant NAC or NADPH oxidase inhibitor apocynin largely suppressed the cytotoxic action of acrolein (Fig. 3D and E). These observations indicate a mediating role of oxidative stress in acrolein‐initiated cell injury.

Given that p38 MAP kinase mediates many oxidative stress‐induced cell responses in a variety of cell types initiated by many insults, including acrolein 32, 33, we tested the role of p38. Exposure of cells to acrolein led to a time‐dependent activation of p38 (Fig. 3F). Inhibition of p38 with SB203580 prevented the appearance of PI‐positive cells (Fig. 3G) and reduced the cellular release of LDH (Fig. 3H), suggesting a pivotal role of p38 in acrolein‐induced urothelial cell injury.

**Figure 4:**
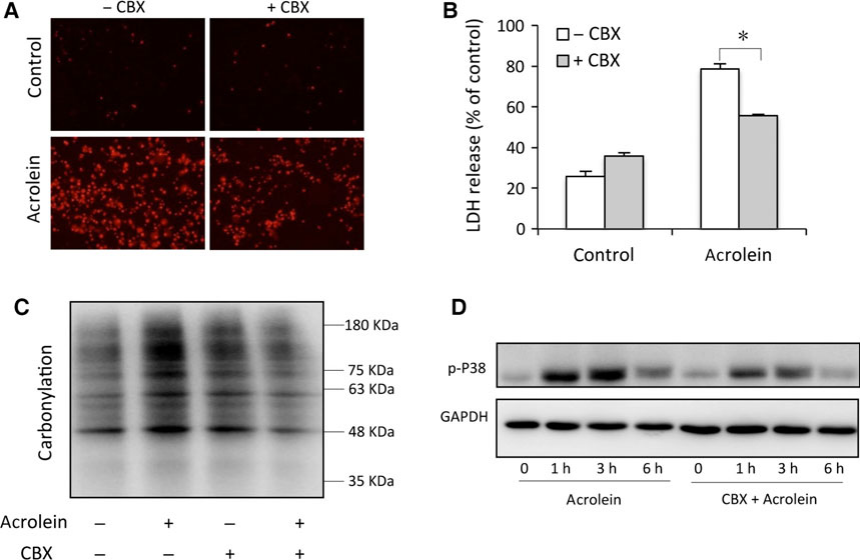
CBX reduces acrolein‐induced cell injury through inhibition of oxidative stress and P38 activation. (**A**–**B**) Effects of CBX on cell viability. Urothelial cells were incubated with 100 μM acrolein for 12 hrs in the presence or absence of 10 μM CBX. Then, the cells were stained with PI (**A**) and cell supernatants were collected for LDH assay (**B**). Data are expressed as mean ± S.E. (*n* = 3). *P < 0.01. (**C**–**D**) Effect of CBX on acrolein‐induced protein carbonylation and P38 activation. Urothelial cells were exposed to 100 μM acrolein for 3 hrs in the presence or absence of CBX. Then, cell lysates were assayed for carbonylated proteins (**C**) and P38 phosphorylation (**D**).

To determine whether and how CBX protected cells against acrolein‐elicited cell injury *in vitro*, we examined the effect of CBX. Figure 4A shows that CBX treatment decreased the number of PI‐positive cells and the level of extracellular released LDH (Fig. 4B). The protective effect of CBX was associated with an obviously reduced level of protein oxidation and p38 phosphorylation (Fig. 4C and D). These observations suggest that CBX alleviates acrolein‐induced cell injury through inhibition of oxidative stress.

### TRPV4 contributes to acrolein‐induced oxidative stress and cell injury in the cultured mouse urothelial cells

**Figure 5:**
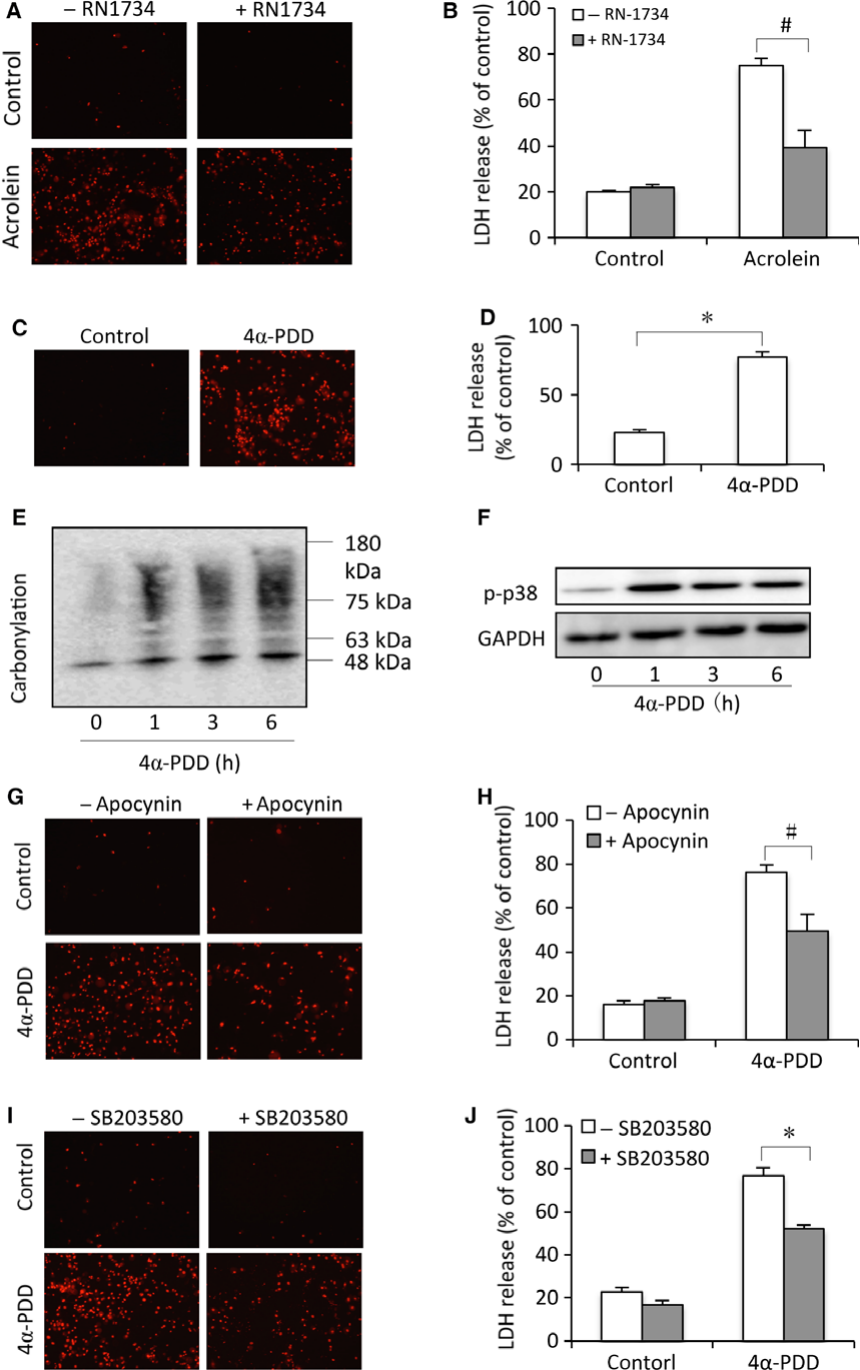
TRPV4 induces oxidative cell injury. (**A**,** B**) Attenuation of acrolein‐induced cell injury by TRPV4 antagonist. Primary urothelial cells were exposed to 100 μM acrolein in the absence or presence of 10 μM RN‐1734 for 24 hrs. Afterwards, the cells were stained with PI (**A**) and cell supernatants were collected for LDH assay (**B**). Data are expressed as mean ± S.E. (*n* = 3). (**C**,** D**) Induction of urothelial injury by the TRPV4 agonist. Urothelial cells were exposed to 10 μM 4α‐PDD for 24 hrs. (**E**,** F**) Induction of protein carbonylation and P38 activation by the TRPV4 agonist. Cells were exposed to 10 μM 4α‐PDD for indicated time intervals. Then, cell lysates were subjected to Western blot analysis for carbonylated proteins (**E**) and P38 phosphorylation (**F**). (**G**,** H**) Effects of NADPH oxidase inhibitor on cell viability. Urothelial cells were exposed to 10 μM 4α‐PDD for 12 hrs in the presence or absence of 10 mM apocynin. Afterwards, cells were stained with PI (**G**) and assayed for LDH release (**H**). (**I**,** J**) Attenuation of 4α‐PDD‐induced cell injury by the P38 inhibitor. Primary urothelial cells were exposed to 10 μM 4α‐PDD in the presence or absence of 20 μM SB203580 for 12 hrs. ^#^ *P* < 0.05. *P < 0.01.

Given that TRPV4 played an important role in CYP‐induced mouse cystitis 9, we, therefore, speculated that TRPV4 might be implicated in acrolein‐induce urothelial cell injury. To test this hypothesis, we examined the influence of TRPV4 inhibition on acrolein‐induced cell injury. Figure 5 shows that inhibition of TRPV4 with RN‐1734 attenuated acrolein‐induced cell injury (Fig. 5A and B). Consistently, activation of TRPV4 with TRPV4 agonist 4α‐PDD‐induced cell injury (Fig. 5C and D), which was associated with increased protein carbonylation and p38 activation (Fig. 5E and F). Intriguingly, inhibition of oxidative stress with NADPH inhibitor apocynin or suppression of p38 with SB203580 also protected cells from TRPV4 agonist‐induced cell injury (Fig. 5G–J).

Of note, the similar results were also obtained from cells treated with another TRPV4 agonist GSK1016790A (Fig. S2). Furthermore, downregulation of TRPV4 with siRNA also attenuated acrolein‐elicited oxidative cell injury in rat tubular epithelial NRK‐E52 cells (data not shown). These observations indicate that TRPV4 activation contributes to acrolein‐initiated activation of P38, induction of oxidative stress, as well as cell injury.

### CBX suppressed TRPV4 agonist‐initiated oxidative cell injury and increase in intracellular Ca^2+^

**Figure 6:**
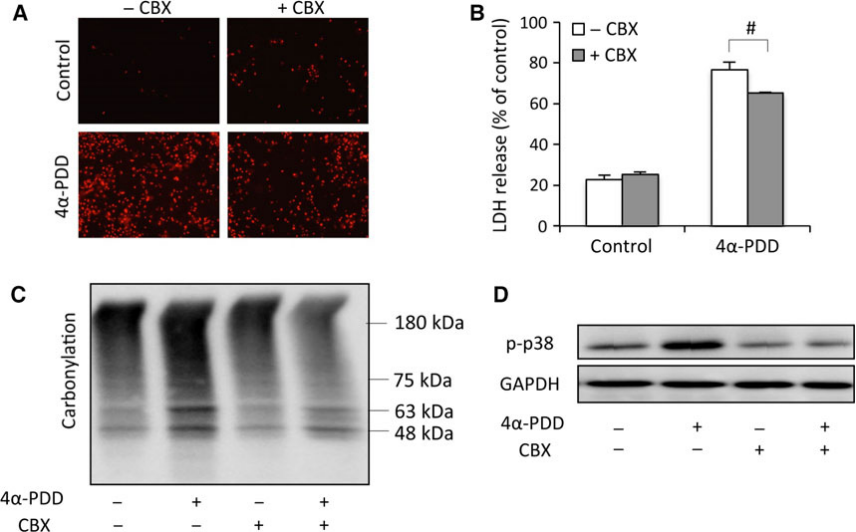
CBX attenuated TRPV4‐mediated oxidative responses. (**A**–**B**) Primarily cultured urothelial cells were exposed to 10 μM 4α‐PDD in the presence or absence of 10 μM CBX for 12 hrs. Afterwards, the cells were stained with PI (**A**), and cell supernatants were collected for LDH assay (**B**). Data are expressed as mean ± S.E. (*n* = 3). ^#^ *P* < 0.05. (**C**–**D**) Effect of CBX on 4α‐PDD‐induced protein carbonylation and P38 activation. Urothelial cells were exposed to 10 μM 4α‐PDD in the presence or absence of CBX for 6 hrs. Cell lysates were assayed for carbonylated proteins (**C**) and p38 phosphorylation (**D**).

We next proceeded to determine whether CBX affected TRPV4‐induced cell responses. Consistent with its protective actions on acrolein, CBX also significantly alleviated the cytotoxicity of 4α‐PDD (Fig. 6A and B). Consistently, CBX suppressed TRPV4‐induced protein oxidation and p38 activation (Fig. 6C and D). These observations indicate that CBX prevents TRPV4‐elicited oxidative cell injury.

**Figure 7:**
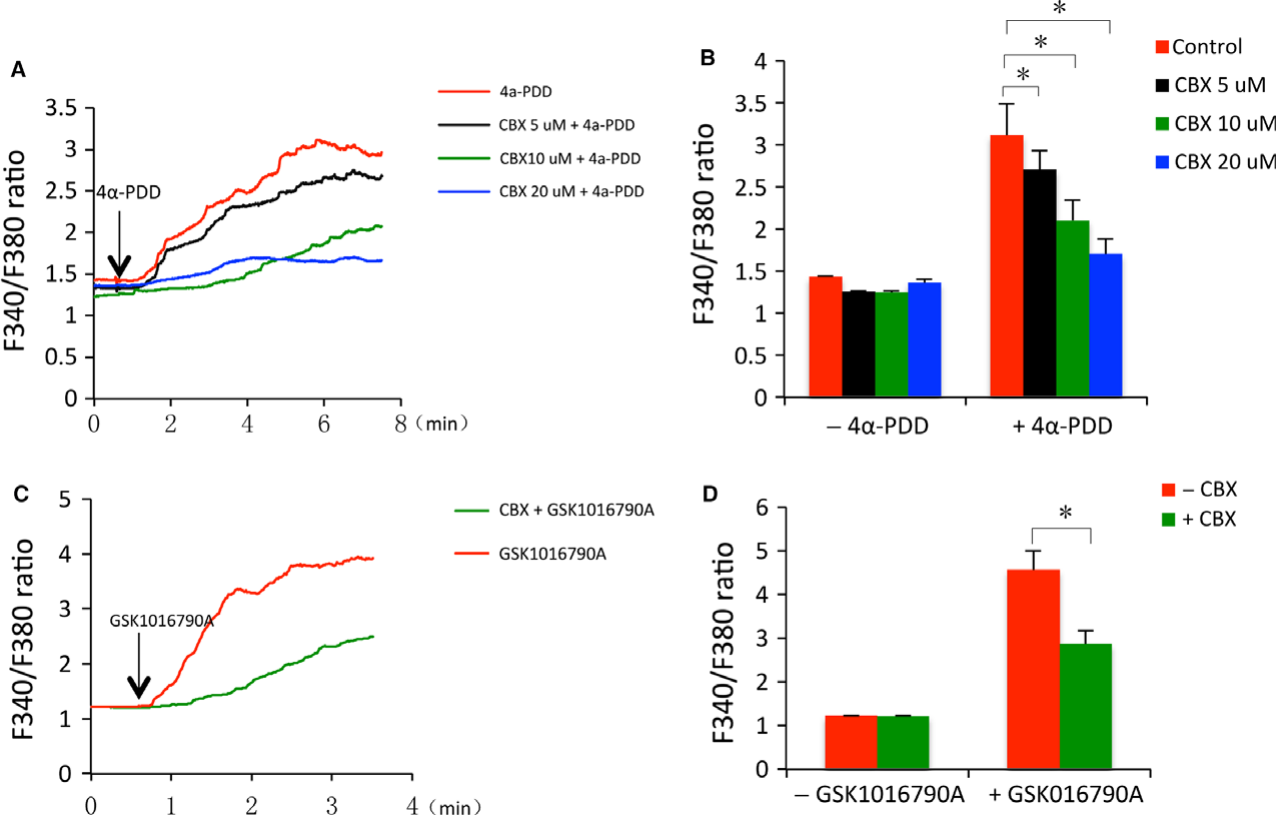
CBX suppresses TRPV4 agonists‐induced increase in intracellular Ca^2+^. (**A**) Effect of CBX on 4α‐PDD‐elicited Ca^2+^ influx. NRK‐52E cells were pre‐treated with or without CBX for 30 min. and exposed to 3 μM 4α‐PDD in the presence or absence of CBX for the indicated time. The average levels of intracellular Ca^2+^ among 80–100 cells in a single study were assessed through ratiometric imaging with fura‐2 at 340 nm and 380 nm (F340/F380). The results are presented as dynamic traces of Ca^2+^ over time. (**B**) The intracellular Ca^2+^ level at basal and peak in (**A**) was quantitated. (**C**) Effect of CBX on GSK1016790A‐elicited Ca^2+^ influx. NRK‐52E cells were either pre‐treated with 10 μM CBX or left untreated for 30 min. and exposed to 5 nM GSK1016790A in the presence or absence of CBX for the indicated time. The intracellular Ca^2+^ measurement was performed the same as above. (**D**) The basal and peak intracellular Ca^2+^ level in (**C**). Data are expressed as mean ± S.E. (*n* = 80 ~ 100). *P < 0.01.

The suppressive effect of CBX on TRPV4‐mediated oxidative responses led us to examine the possible influence of CBX on TRPV4 channel activity. Given that TRPV4 activation causes Ca^2+^ influx, we, therefore, examined the effect of CBX on TRPV4 agonist‐elicited changes in Ca^2+^. As expected, stimulation of cells with 4α‐PDD caused a rapid and robust increase in intracellular Ca^2+^ (Fig. 7A and B). In the presence of CBX, however, the Ca^2+^ increase was blunted. This effect of CBX was concentration‐dependent and was also observed in Ca^2+^ response triggered by another TRPV4 agonist GSK1016790A (Fig. 7C and D). These results indicate that CBX inhibits TRPV4 channel activities.

## Discussion

In this study, we demonstrated that CBX prevented acrolein‐initiated urothelial cell injury *in vitro* and improved CYP‐induced bladder dysfunction *in vivo*. Furthermore, we revealed that the effect of CBX could be attributed to its inhibitory effect on TRPV4 channels. Our study thus provides novel mechanical insights into the pharmacologic actions of CBX and suggests that CBX might be a promising agent for treatment of voiding dysfunction.

We have used mouse CYP cystitis to investigate the therapeutic effects of CBX on bladder dysfunction. CYP is a chemotherapy drug for treatment of cancer and autoimmune diseases. One of the side effects of CYP is to cause cystitis, as manifested by histologic changes in bladder and frequent voiding 31, 34, 35. Using mouse model of CYP cystitis, we demonstrated that CBX effectively ameliorated CYP‐induced bladder protein oxidation, inflammation and improved bladder function, indicating that CBX has the therapeutic potential for voiding dysfunction.

CYP‐induced cystitis has been reported to be the consequence of the accumulation of its metabolic product acrolein in urothelium 21, 30, 31. As a reactive aldehyde, acrolein rapidly depletes intracellular GSH and inhibits thioredoxin, weakening the major cellular defence system against oxidative stress. Also, acrolein promotes superoxide anion production through activation of NADPH oxidase 33, 36. These molecular events could also occur in our system. Indeed, acrolein‐induced protein oxidation, p38 activation, and urothelial injury were alleviated by antioxidant NAC and NADPH oxidase inhibitor apocynin. The similar protective effect of CBX on these oxidative responses implies that CBX may work through interruption of acrolein‐induced oxidative stress.

What are the possible molecules targeted by CBX? Because CBX is a multiple channel inhibitor, we have focused on its action on channels, especially TRPV4. Previous studies have shown that genetic disruption or pharmacological inhibition of TRPV4 improves CYP‐induced bladder dysfunction 9, 11. Using an *in vitro* model, we demonstrated a direct participation of TRPV4 in acrolein‐induced oxidative responses. Currently, it is unclear whether acrolein directly activated TRPV4 or indirectly after its induction of oxidative stress. We favoured the latter possibility because our preliminary experiments showed that, in comparison with TRPV4 agonist, acrolein only induced a weak Ca^2+^ response (data not shown). Recently, activation of TRPV4 channels by oxidative stress and participation of TRPV4 in oxidative stress‐induced cell death have been reported 37, 38, 39. The prolonged activation of Ca^2+^‐permeable TRPV4 channels may lead to Ca^2+^ overload, causing oxidative stress, and cell injury 40. Increased Ca^2+^ causes oxidative stress through multiple mechanisms and has been shown to be linked to cell injury induced by different insults, including acrolein 41. Our preliminary experiment showed that the cell death initiated by TRPV4 agonist was exaggerated by A23187, a Ca^2+^‐ionophore, and attenuated by U73122 (an inhibitor of phospholipase C that plays a key role in Ca^2+^‐mediated responses), supporting a critical involvement of Ca^2+^ (data not shown). Intriguingly, CBX potently suppressed TRPV4‐mediated Ca^2+^ influx, suggesting that the cytoprotective effect of CBX may be through direct inhibition of TRPV4 channels.

Of note, apart from the suppressive effects on TRPV4, CBX could also exert its protective effects through inhibition of Cx, pannexin or other channels. Participation of these channels in CYP cystitis has been well documented. We and others have shown that the increased Cx43 contributed to the altered micturition pattern in mouse CYP cystitis 5, 22. Okinami *et al*. 22 reported that treatment of mouse with gap junction inhibitor 18α‐glycyrrhetinic acid alleviated frequent voiding. Recently, we have found that oxidative stress activated Cx43 hemichannels, which, in turn, contributed to the disassembly of cell junctions and cell injury through mechanisms involving oxidative stress and p38 42, 43. Similar to Cx channels, a pivotal role of pannexin‐1 channels in the induction of bladder inflammation and voiding dysfunction has also been described 6, 44. Intriguingly, recent studies revealed that there is a complicated interactive relationship among these channels. For example, pannexin‐1 channels are requisite for inflammatory mediator‐induced Cx43 expression in bladder 6. Activation of TRPV4 led to the opening of Cx and pannexin channels, which mediated some of the biological actions of TRPV4 45, 46, 47. In this context, the therapeutic effect of CBX observed in this study could be the result of its inhibitory actions on multiple channels.

It is also worth mentioning that the suppression of Ca^2+^ signalling by CBX could also be ascribed to its suppressive action on Cx and/or pannexin hemichannels. These channels may transmit, propagate and potentiate TRPV4 agonist‐initiated Ca^2+^ signal through direct intercellular Cx channel or pannexin‐derived extracellular ATP 10, 48, 49, 50. Furthermore, CBX has also been reported to block L‐type voltage‐gated Ca^2+^ channels and prevent the light‐evoked increase in intracellular Ca^2+^ in retinal ganglion cells and cultured rat cortical astroglia, respectively 51, 52. Intriguingly, this channel has also reported to be implicated in diabetic bladder dysfunction 53. It appears that CBX may influence Ca^2+^ signal induced by multiple channels in the bladder.

Our findings could have important basic and clinic implications. First, our study provides novel mechanistic insight into CYP‐induced cystitis. We characterized TRPV4 channels, oxidative stress and p38 activation as important molecular events behind acrolein‐induced urothelial cell injury. Therapeutic strategies against these events could be developed for treatment of urothelial cell injury. Second, our study expands our understanding about the pharmacologic actions of CBX. Our results show that CBX may directly inhibit TRPV4 channels. This property of CBX might be exploited for the treatment of certain diseases in which TRPV4 plays a pivotal role, such as bronchitis and colitis 54, 55. Conversely, cautions should be taken in explanation of the results obtained from CBX, when used as a single channel inhibitor in pharmacological and physiological research. Validation of the results using more specific inhibitors may be necessary. Third, our study suggests that CBX might have great advantages over other channel inhibitors in the treatment of bladder dysfunction because of its potency in inhibition of multiple channels, clinical availability and safety. Of note, our study also has limitations. The mouse model used in this investigation is featured by dramatic changes in bladder morphology, pronounced oedema, obvious urothelial injury, as well as oxidative and inflammatory cell responses, which are far more severe than human pathology in the majority of OAB patients. Furthermore, the results of this study were obtained within 24 hrs after CYP administration, whereas in the clinic, OAB is a chronic health problem. Moreover, the role of TRPV4 channels in OAB patients has not been firmly established. It remains to be seen whether CBX could also be applied to treat voiding dysfunctions in the clinic.

Collectively, CBX effectively attenuates CYP‐induced mouse voiding dysfunction, possibly through mechanisms involving suppression of TRPV4 channels and the channel‐initiated oxidative stress. Our study provides novel mechanistic insights into pharmacological actions of CBX and suggests that CBX might be a promising agent for treatment of bladder dysfunction.

## Conflict of interest

The authors confirm that there are no conflicts of interest.

## Supporting information


**Figure S1** Representative historical changes of bladder from CYP‐treated mouse

**Figure S2** TRPV4 agoinst GSK1016709A induces oxidative cell injury in primarily cultured urothelial cells
