# Supplementary figures and images for: Carbenoxolone inhibits TRPV4 channel‐initiated oxidative urothelial injury and ameliorates cyclophosphamide‐induced bladder dysfunction
Source: J Cell Mol Med. 2017 Feb 28;21(9):1791–802. doi: 10.1111/jcmm.13100 (PMC5571544; doi:10.1111/jcmm.13100)

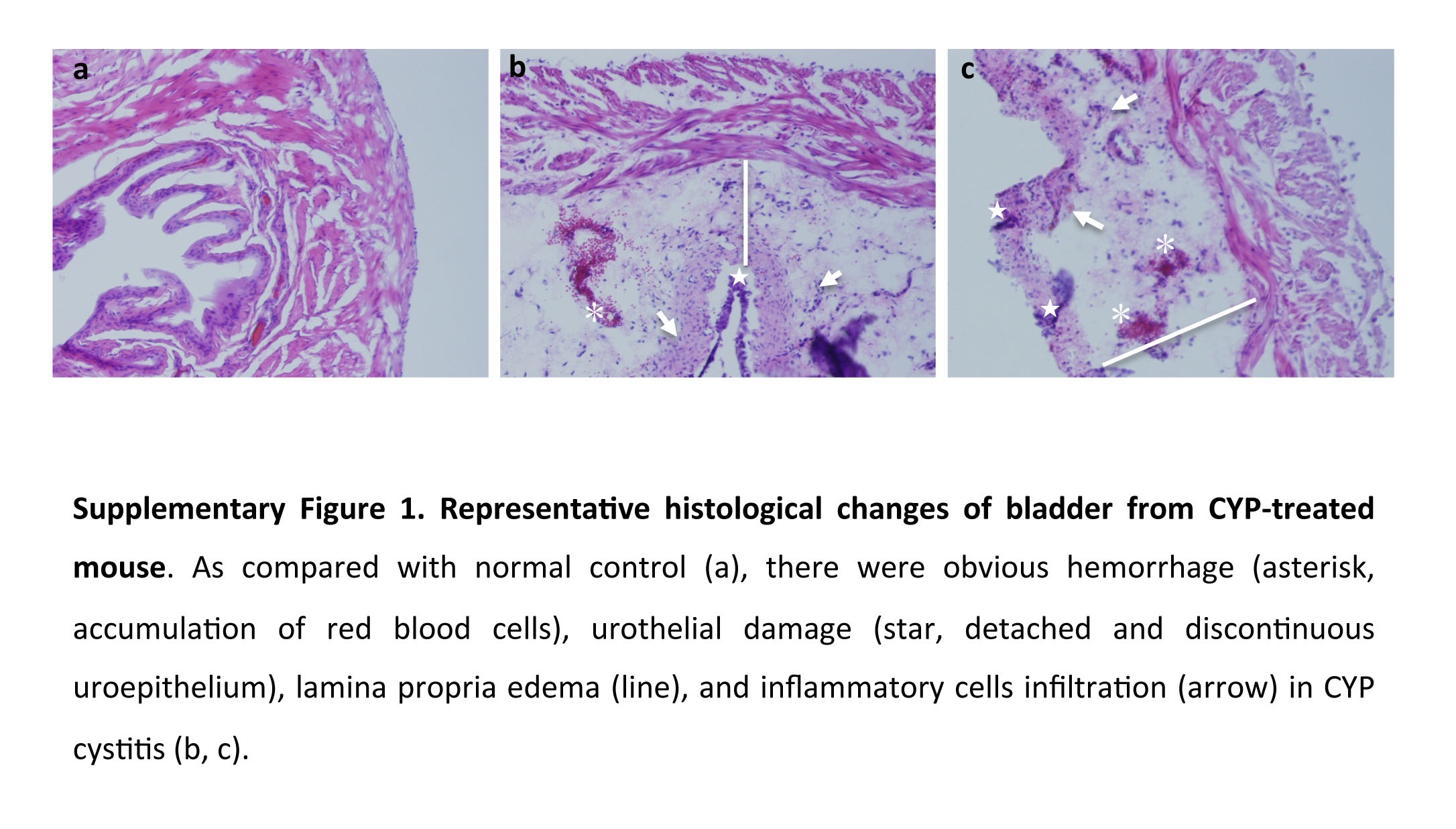

Supplement: Supplementary file 1 — Figure S1 Representative historical changes of bladder from CYP‐treated mouse [file JCMM-21-1791-s001.jpg]

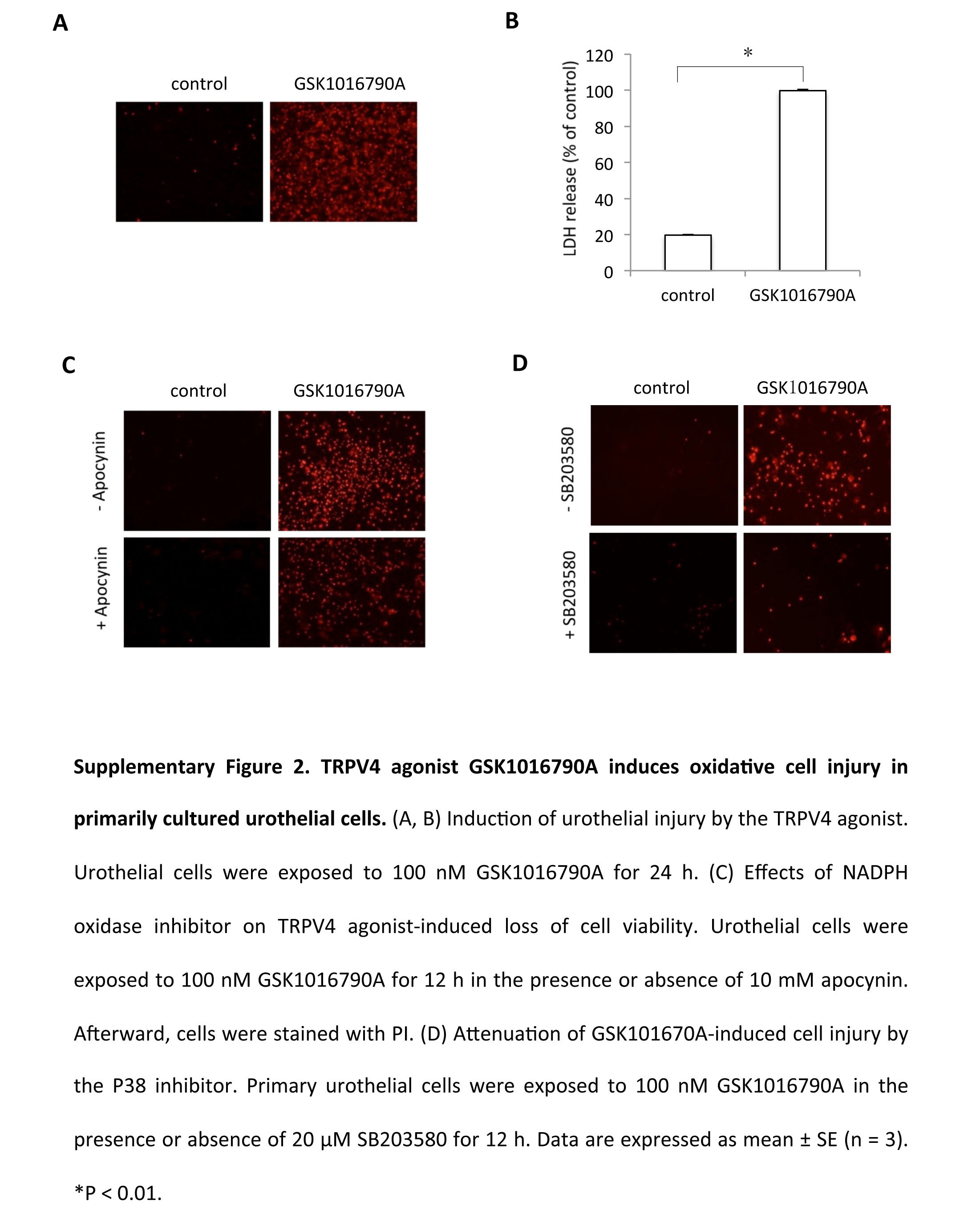

Supplement: Supplementary file 2 — Figure S2 TRPV4 agoinst GSK1016709A induces oxidative cell injury in primarily cultured urothelial cells [file JCMM-21-1791-s002.jpg]
